# Evolution of the Serendipitous Discovery of Macrophage–Lymphocyte Interactions

**DOI:** 10.3389/fimmu.2014.00530

**Published:** 2014-10-27

**Authors:** Joost J. Oppenheim

**Affiliations:** ^1^Laboratory of Molecular Immunology, National Cancer Institute, National Institutes of Health, Frederick, MD, USA

**Keywords:** lymphocytes, dendritic cells, macrophages, antigen presentation, chronic lymphocytic leukemic cells

This contribution to the project on the Living History of Immunology concerning “The Transformation of Column-Purified Lymphocytes with Non-specific and Specific Antigenic Stimuli,” by Joost J. Oppenheim, Brigid G. Leventhal, and Evan M. Hersh represents another excellent example of research based on a serendipitous discovery snatched from the jaws of a failed project (1). Evan, Brigid, and I were all clinical associates at the NCI engaged in the care of patients with leukemia and solid tumors from 1962 until 1965. As a reward for our clinical efforts, we were given the opportunity to pursue laboratory research studies with one of the principal investigators for the last 2 years of our stay.

Following several false starts, I ended up in the laboratory of Dr. Jacqueline Wang Peng, who was an expert in studies of chromosome abnormalities caused by neoplastic changes and damage from chemotherapeutic and radiation treatments. Our chromosome analyses were frustrated by the failure of leukemic lymphocytes from chronic lymphocytic leukemia (CLL) patients to be activated to divide and develop metaphases that could be analyzed for chromosome breaks in response to a kidney bean extract known as phytohemagglutinin (PHA). Since the peripheral blood (PB) of more advanced CLL patients contained high numbers of white blood cells (WBC) consisting entirely of lymphocytes, we decided as a control to purify the non-adherent lymphocytes present in normal PBWBC by eluting them off sterile glass bead or nylon fiber columns, which retained the adherent phagocytic neutrophils and monocytes.

After numerous mishaps and considerable practice, these columns yielded at least 98% pure lymphocytes based on microscopic analysis. We were dismayed to find that these purified normal lymphocytes were also hyporesponsive to a variety of antigenic stimulants such as tetanus toxoid and streptolysin O, but still showed normal proliferative response to the more potent polyclonal PHA stimulant. This was determined from the proportion of cells undergoing morphological blastogenesis and the uptake of tritiated thymidine. However, the purified lymphocytes in comparison with unpurified normal lymphocytes were also hyporesponsive to suboptimal doses of PHA. Of course, we were very concerned that the column procedure had damaged the cells, but we failed to observe any evidence of cell death based on trypan blue uptake. Furthermore, when cultured at a higher cell density, the lymphoproliferative response to antigens showed some recovery arguing against cell damage. This observation also suggested the possibility that the few residual contaminating non-lymphocytic cells might be interacting more effectively over the shorter distances at higher cell densities. We tested this idea by adding some unfractionated WBC back to the cultures of purified lymphocytes, which partially restored the lymphoproliferative response to antigens. A feeder layer consisting of WI-38 human embryonic fibroblasts had no restorative effect.

These results unfortunately failed to shed any light on the unresponsiveness of CLL cells. However, they pointed to the requirement for a cooperative interaction between phagocytic cells and lymphocytes. Based on the available literature, we proposed that macrophages were somehow facilitating the activation of lymphocytes to “transform” and proliferate. Evan Hersh and Jules Harris obtained convincing evidence in support of this hypothesis by restoring the lymphoproliferative responses by the addition of coverslips with adherent human macrophages to the cultures of purified lymphocytes (2).

I further pursued my immunological studies during a sabbatical year at the University of Birmingham in England from 1965 to 1966, where I learned to work with non-human species and showed that purified lymphocytes from guinea pig lymph nodes were also unresponsive to antigenic stimulants unless supplemented with some phagocytic cells. Upon returning to the Dental Institute at the National Institutes of Health (NIH), I was joined by a pediatrician, Dr. Robert Seeger in investigations of the role of macrophages in immunity. We were able to show that footpad injection of peritoneal macrophages from syngeneic guinea pigs after a brief exposure to antigens such as ovalbumin induced greater delayed hypersensitivity (DTH) reactions and were better at priming antibody responses than equal or higher doses of soluble antigens (3). Furthermore, antigens were taken up much less well by lymphocytes, thymocytes, and hepatoma cells than to macrophages and these cells were not immunogenic (4). Thus, macrophages could activate T lymphocytes to mediate DTH and prime B-cell antibody production. Bob Seeger and I also determined that peritoneal, alveolar, or PB macrophages obtained from either immune or non-immune donors were all equally effective at priming immune responses (5). However, macrophages could not induce non-immune lymphocytes to proliferate. Thus immune specificity and memory appeared to be a property of lymphocytes rather than macrophages.

In the course of our studies, Bob and I noticed that syngeneic macrophages were much more effective than allogeneic macrophages, but we did not pursue this issue. Alan Rosenthal thoroughly investigated the role of histocompatibility in this interaction. Alan Rosenthal and Ethan Shevach went on to show that the macrophage–lymphocyte interactions required MHC compatibility to be successful (6). They further determined, using alloantisera against MHC antigens, that macrophage MHC was necessary for T lymphocytic recognition of antigens (7). Of course, Ralph Steinman’s discovery that dendritic cells (DC) contaminating the macrophage preparations were actually the most potent antigen presenting cells superseded our findings (8). However, I must confess that I found it difficult to accept the idea that the small contaminant population of DC rather than macrophages was responsible for antigen presentation, until this became incontrovertible based on the *in vitro* studies of Jacques Banchereau and his colleagues (9). They were able to produce large numbers of dendritic/Langerhans cells *in vitro* by culturing cord blood hematopoietic progenitor cells with a combination of granulocyte-macrophage colony stimulating factor and tumor necrosis factor. These cells had the morphology and phenotypic markers of DC and were very potent at presenting antigens and priming T lymphocytes.

Our serendipitous finding that T cells require accessory cells for antigen presentation was based on an initial desire to better understand the failure of lymphocytes from CLL patients to transform in response to stimulation. Other investigators have determined that CLL cells are usually monoclonal B lymphocytes that do not respond to T cell stimulants (10). This was followed by curiosity on our part to better understand the inability of purified normal peripheral human lymphocytes to respond to antigenic stimulation unless supplemented by macrophages. Our observations led other investigators to discover the crucial role of MHC in antigen presentation, antigen processing, and the outstanding capacity of DC to activate T cell-dependent immune responses. These consequent findings went beyond the scope of our imagination. In conclusion, our unexpected scientific findings clearly contributed in an unanticipated manner to a greater understanding of adaptive immunity and clearly illustrate the stepwise communal process of scientific progress.

## Conflict of Interest Statement

The author declares that the research was conducted in the absence of any commercial or financial relationships that could be construed as a potential conflict of interest.
